# A systematic review of interventions to improve uptake of pertussis vaccination in pregnancy

**DOI:** 10.1371/journal.pone.0214538

**Published:** 2019-03-28

**Authors:** Hassen Mohammed, Mark McMillan, Claire T. Roberts, Helen S. Marshall

**Affiliations:** 1 Adelaide Medical School and Robinson Research Institute, University of Adelaide, Adelaide, South Australia, Australia; 2 Vaccinology and Immunology Research Trials Unit (VIRTU), Women’s and Children’s Hospital, North Adelaide, South Australia, Australia; 3 School of Public Health, University of Adelaide, Adelaide, South Australia, Australia; Public Health England, UNITED KINGDOM

## Abstract

**Background:**

Maternal pertussis vaccination has been introduced in several countries to prevent pertussis morbidity and mortality in infants too young to be vaccinated. Our review aimed to systematically collect and summarize the available evidence on the effectiveness of interventions used to improve pertussis vaccination uptake in pregnant women.

**Methods:**

We conducted a systematic search of MEDLINE/PubMed, PMC and CINAHL. Before and after studies and those with a concurrent control group were considered for inclusion. Standardized effect sizes were described as the ratio of the odds to be vaccinated in the intervention group compared with the standard care group and absolute benefit increase (ABI) were calculated.

**Results:**

Six studies were included in the review, of which three were randomized controlled trials (RCTs). Strategies to improve uptake were focused on healthcare providers, pregnant women, or enhancing vaccine access. Healthcare provider interventions included provider reminder, education, feedback and standing orders. Interventions directed at pregnant women focused solely on education. Observational studies showed: (1) the provision of maternal pertussis vaccination by midwives at the place of antenatal care has improved uptake of pertussis vaccine during pregnancy from 20% to 90%; (2) introduction of an automated reminder within the electronic medical record was associated with an improvement in the pertussis immunization rate from 48% to 97%; (3) an increase in prenatal pertussis vaccine uptake from 36% to 61% after strategies to increase provider awareness of recommendations were introduced. In contrast to these findings, interventions in all three RCTs (2 involved education of pregnant women, 1 had multi-component interventions) did not demonstrate improved vaccination uptake.

**Conclusions:**

Based on the existing research, we recommend incorporating midwife delivered maternal immunization programs at antenatal clinics, use of a provider reminder system to target unvaccinated pregnant women and include maternal pertussis immunization as part of standard antenatal care.

## Introduction

There has been a global resurgence in pertussis in recent years, particularly in the US, the UK and Australia, with the highest rates of hospitalization and death in young infants, mainly those less than 2 months of age, prior to the recommended age for vaccination [1–6]. Infection of young infants occurs primarily at the household level with new mothers identified as the most common sources [7, 8]. Maternal pertussis immunization protects infants through passive and active transfer of maternal antibodies that protect the infant until the primary immunization series commences in infants at 6–8 weeks of age [9–11]. The highest level of protection is not achieved in infants until they have received 3 doses at 6 months of age [12]. Pertussis vaccination in pregnancy at least 7 days before delivery can prevent up to 91% of pertussis disease in infants age <3 months [11]. In 2011, the US became the first country to recommend that health care personnel administer pertussis vaccine to pregnant women [13] and many countries have recently adopted this policy in an attempt to reduce the burden of pertussis in young infants [14]. Despite the recommendation of maternal pertussis vaccination from immunization advisory groups internationally [13–15], uptake remains suboptimal [16–19]. The barriers to vaccination in pregnancy are more complicated than the barriers identified for low uptake in childhood immunization programs [20].

Some recent studies have evaluated the effectiveness of strategies in improving maternal immunization uptake, which predominantly focussed on educational interventions for pregnant women or healthcare providers while others included a multi-component intervention package [21–25]. A systematic review has been recently published to identify effective strategies in improving the uptake of vaccination in pregnancy in high-income countries [26]. However, the review [26] was aimed to make recommendations to an English setting and the majority of the published articles (18/22) identified in the review evaluated the effectiveness of strategies in improving seasonal influenza vaccination uptake in pregnancy. Limited data exist on rigorously evaluated interventions to improve pertussis immunization uptake among pregnant women. Given the well-documented benefits of maternal pertussis immunization in protecting very young infants, determining effective strategies to improve pertussis vaccine uptake during pregnancy should be a public health priority. This is the first review aimed to systematically collect and summarize the available evidence on the effectiveness of interventions in improving pertussis vaccination uptake in pregnant women. The protocol for this review is published in PROSPERO International prospective register of systematic reviews—CRD42017058178.

## Materials and methods

This systematic review was conducted in accordance with the Preferred Reporting Items for Systematic Reviews and Meta-Analyses (PRISMA) statement (see S1 Table) [27].

### Search strategy

The search strategy included the following electronic databases:—PubMed, PMC, Medline, Cochrane Library, CINAHL and ClinicalTrials.gov. Other sources include conference proceedings—World Society for Paediatric Infectious Diseases (WSPID) and European Society for Paediatric Infectious Diseases (ESPID). Specific search terms suitable to the individual databases were developed. These search terms included combinations of Medical Subject Headings (Messi)/Emtree and text words contained in the title and abstract (see S2 Table).

### Eligibility criteria

Our systematic review includes all original studies that reported on interventions to improve pertussis uptake during pregnancy. Some countries recommending pertussis vaccination during pregnancy are using combined tetanus toxoid, reduced diphtheria toxoid, and acellular pertussis (Tdap) or with inactivated polio vaccine (Tdap-IPV) in their programs. Hence, studies comparing pertussis vaccination uptake in pregnancy combined with or without other antigens either pre-post introduction of intervention or a concurrent control group during the same observation period were considered. The primary outcome measured was pertussis vaccination uptake during pregnancy, with confirmation in electronic medical records or self-reported data (Table 1).

**Table 1:** The inclusion and exclusion criteria used during the screening process.

Criteria	Included
Study design	Studies comparing pertussis vaccine uptake among pregnant women who were exposed to an intervention vs. standard care Observational studies Randomised controlled trials Interventions that include pertussis as a compound of the immunization i.e. Tdap or Tdap-IPV
Population	Pregnant women
Outcomes	Pertussis vaccination uptake during pregnancy (Standard care vs. intervention group)
Publication date	Up to January 2019
Language	Studies published in English

### Study selection

Two independent reviewers (HM and MM) completed initial screening based on titles and abstracts of potentially relevant studies. If the articles reported interventions to improve pertussis vaccination uptake during pregnancy, the reviewers performed a more detailed subsequent assessment by looking at the full text. The reference lists considered for inclusion were searched for additional studies that might have been missed in the database search. Disagreements about the inclusion or exclusion of studies were resolved through consensus discussions among reviewers.

### Data analysis

The primary measures extracted were percentage changes in uptake of pertussis vaccination during pregnancy from standard care group to intervention group. Standardized effect sizes were described as the ratio of the odds to be vaccinated in the intervention group compared with the standard care group and absolute benefit increase (ABI) with 95% confidence intervals (CI), were calculated. In studies with concurrent comparison groups, the overall change in pertussis vaccination uptake was calculated by using the difference in vaccine uptake change observed in the intervention and comparison groups. In studies without a concurrent comparison group, the absolute percentage change was calculated from measurements of pertussis vaccination uptake during pregnancy in pre- and post-intervention. Additionally, a list of all confounders adjusted for in the data analysis was reported. To strengthen the generalisability of our review results, we used the intervention classification guidelines adopted from the Task Force on Community Preventive Services [28].

1. Provider-focused interventions
2. Pregnant woman-focused interventions
3. Interventions to enhance maternal pertussis vaccination access

Our review did not conduct meta-analysis because of the broad heterogeneity in study design and types of interventions used to improve pertussis vaccination uptake during pregnancy.

### Data quality assessment

Two independent reviewers (HM and MM) assessed the quality of the included studies. The Cochrane Collaboration method was used for the risk of bias assessment of randomized controlled trials (RCTs) [29]. The risk of bias was assessed in six domains: sequence generation, allocation concealment, blinding, incomplete outcome data, selective outcome reporting and ‘other issues'. A ‘risk of bias summary’ displaying the quality assessment of all included RCT studies was generated. For each outcome, the Grading of Recommendations Assessment, Development and Evaluation (GRADE) criteria were also used to evaluate the quality of the RCT studies [30]. The GRADE criteria were used along with the Cochrane Collaboration tool because these criteria, take into account assessment of three additional domains: consistency, directness, and precision of the results in addition to the risk of bias. Randomized trials began as high-quality evidence but were rated down if trials demonstrated limitations (see S3 Table). The Joanna Briggs Institute (JBI) critical appraisal tools were used to assess the quality of experimental studies without random allocation (observational studies) (see S4 Table) [31].

## Results

### Search results

**Fig 1:**
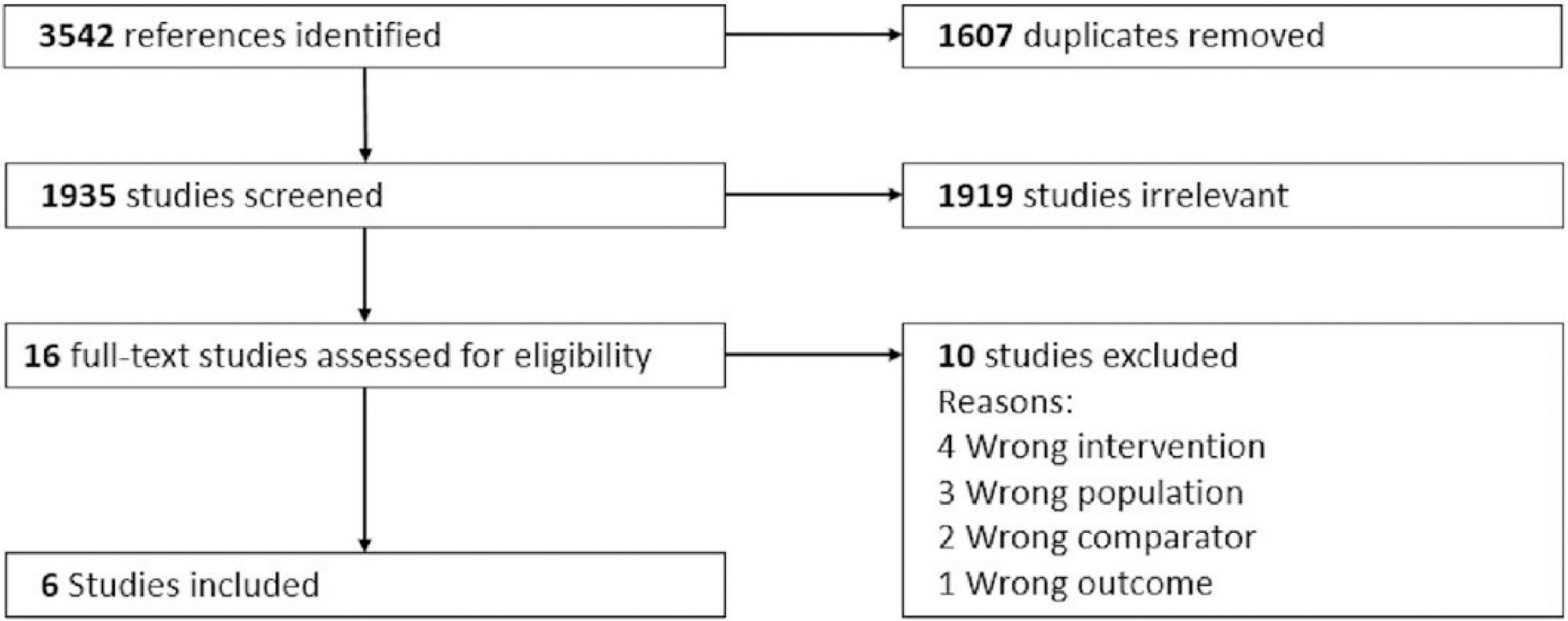
Flow diagram of the process and results of study selection.

The initial search generated 3542 published studies. After removing duplicates, screening titles and abstracts of the remaining 1935 studies, 16 studies were identified for full text review (Fig 1). Of these, we excluded 10 papers because they did not include an intervention component (n = 4), eligible population (n = 3), outcome of interest (n = 1) or did not have a standard care group for comparison (n = 2) (see S5 Table). Six studies that met the selection criteria were included. No additional studies were obtained from the reference lists of the included studies.

### Study characteristics

The six included studies were published between 2015 and 2017. Five studies were conducted in the United States (US) [23–25, 32, 33] and one study was conducted in Australia [34]. The sample sizes varied from 106 to 10,600 participants. Pregnant women were recruited from public maternity hospitals, tertiary hospitals, antenatal clinics, university hospitals and a multispecialty medical organization.

The studies investigated a variety of interventions; two studies used provider-based interventions only [25, 32], two studies used pregnant woman-focused interventions only [24, 33] and two studies incorporated provider-focused interventions, pregnant woman-focused interventions, as well as interventions to enhance maternal pertussis vaccination access (Table 2) [23, 34]. Standard care varied and included pre-intervention routine prenatal care [23, 24, 32, 34], routinely offered pertussis vaccination only during the postpartum period [25] and standard Vaccine Information Statements (VISs) produced by the Centers for Disease Control and Prevention (CDC) [33].

**Table 2:** Strategies used to improve pertussis vaccination uptake among pregnant women.

Included studies	Interventions for health care providers				Pregnant women focussed intervention	Interventions to enhance vaccination access	
	Provider reminder/recall	Provider Education	Standing orders	Provider feedback	Pregnant women education	Extend service location	Increase stock
Kriss [24]					√		
Payakachat [33]					√		
Chamberlain [23]		√		√	√	√	√
Morgan [25]	√		√				
Healey [32]	√	√	√				
Mohammed [34]		√	√		√		√

### Critical appraisal

#### Randomized controlled trials

The evidence quality of the two RCTs were rated “moderate” [23, 32] and “low” [24]. In two studies, the proportion of missing outcomes likely resulted in bias of the effect estimates [23, 24]. Self-report was the primary method used to judge if a pertussis vaccine was administered in two of the RCTs [23, 24]. In Chamberlain et al. [23] there was a higher proportion of self-reported vaccination in the intervention group compared to the standard care group, which may have introduced bias. Kris et al. [24] assessed the outcome via self-report during a follow-up survey which could introduce recall bias (Supplementary File).

**Fig 2:**
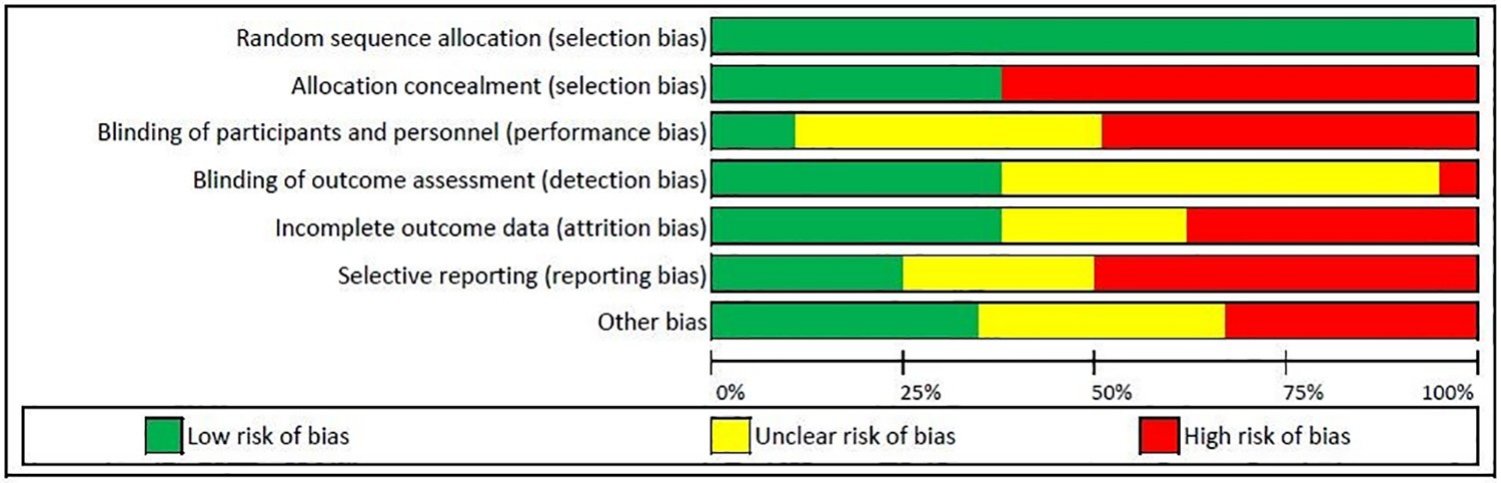
Risk of bias in included RCT studies.

Two of the RCT studies [23, 24] did not have a sufficient number of participants in both arms to achieve 80% power to detect effects caused by the interventions while only one of the RCT studies met the required sample size [33]. One RCT targeted minority women who were African American women [24]. One of the RCT [33] studies was conducted in only one public hospital and the majority of participants were from low socioeconomic backgrounds and had poor health literacy. Hence, these findings may not be representative of other pregnant women in different US regions [33]. The risk of bias of all RCTs is summarized in Fig 2.

#### Observational studies

For all observational studies, interventions were introduced with the aim to improve the uptake of pertussis vaccines among pregnant women. These were assessed using electronic medical records [25, 32] or self-reported data [34]. Two studies [25, 34] included pregnant women who were recruited prior to the recommendation as the standard care groups and the intervention groups included women recruited after the change in the pertussis vaccination recommendations. Hence, observed improvement in vaccination rates could also be attributed to the change in national recommendations in these studies [25, 34]. These observational studies are likely to result in chronology bias and an overestimation of the effect of an intervention. Adjustment for confounding was performed in two of the observational studies (Table 3) [23, 34]. However, not all of the observational studies have considered potential confounders influencing vaccination uptake during pregnancy in their adjusted analysis such as maternal age, parity, primary language, ethnicity, socioeconomic factors, educational level and marital status.

**Table 3:** Absolute benefit increase and 95% confidence intervals of each intervention.

Author	Study design, period and methods	Participants and setting	Uptake of maternal Tdap vaccine (n, %)	Absolute benefit increase, ABI (95% CI)	Confounders adjusted for
**A. Pregnant women focused intervention programs**					
**Payakachat** [33]	**RCT**:	Academic medical centre Arkansas, USA	**Standard care**	**0.03** (-0.07, 0.15)	None
	May–August 2014		65/144 (45%)		
	**Standard care**:				
	Vaccine information statement (sVIS)		**Intervention**		
			66/135 (49%)		
	**Intervention**:				
	Plain language version (mVIS)				
**Kriss** [24]	**RCT**:	Pregnant African American women in 4 antenatal clinics in metropolitan Atlanta, USA	**Standard care**	**Intervention 1**	None
	January 30-April 3, 2013.		2/34 (6%)during pregnancy	**0.00** (-0.13,0.15)	
	Follow up: after delivery		4/34 (12%) postpartum	**Intervention 2**	
	**Standard care**:		**Intervention 1 (Video)**	**0.00** (-0.13, 0.16)	
	Routine prenatal care		2/31 (6%) during pregnancy	**Postpartum**	
	**Intervention**:		7/31 (23%) Postpartum	**Intervention 1**	
	1. Messaging video		**Intervention 2 (iBook)**	**0.10** (-0.07,0.29)	
	2. Messaging iBook		2/30 (7%) during pregnancy	**Intervention 2**	
			13/30 (43%) postpartum	**0.31** (0.09, 0.50)	
**B. Healthcare provider focused intervention programs**					
**Morgan** [25]	**Retrospective study**	Pregnant women from Parkland Hospital, USA	**Standard care**	**0.49** (0.48,0.50)	None
	**Standard care**: Routinely offered Tdap during the postpartum period. Historical control, Jan 2012 to May 2013		5,064/10,600 (48%)		

			**Intervention**		
			9,879/10,201 (97%)		

	**Intervention** A best-practice alert, June 2013 to July 2014				

**Healey** [32]	**Retrospective study**	Women delivering at Texas Children’s Hospital, USA	**Standard care**	**0.25** (0.11,0.37)	None
	**Standard care**: Routine antenatal care. Historical control April to Sept 2013		(36%)^a^		

			**Intervention**:		
	**Intervention**: ACOG “toolkit” Physicians information through email and regular meetings. Sep 2013 to Jun 2014		(61%)^a^		

			N = 6577		

**C. Interventions with bundled components**					
**Mohammed** [34]	**Observational prospective study**	Pregnant women attending a territory obstetric hospital in Adelaide, Australia	**Standard care**	**0.70** (0.50, 0.82)	Age, parity, country of birth, provider recommendation
			5/25 (20%)		
	November 2014 and July 2016				
	**Standard care**: Routine antenatal care		**Intervention**		
			140/155 (90%)		
	**Intervention**: A midwife delivered immunization program				


**Chamberlain** [23]	**A cluster RCT**	Pregnant women from obstetric practices in Georgia, USA	**Standard care**	**0.04** (-0.02,0.12)	Adjusted for clustered study design and intention to receive the vaccine before delivery
	December 2012–April 2013		13/151 (9%)		
	**Standard care** Routine antenatal care				
			**Intervention**		
	**Intervention** Vaccine Champions, provider-to-patient talking points, educational brochures, posters, lapel buttons & iPads loaded with tutorials		19/140 (14%)		
	
	
	
	
	

^a^ The authors did not state the number of vaccinated women pre-and post-intervention

### Effect of various interventions in increasing pertussis vaccine uptake

#### Provider-focused interventions

Two retrospective cohort studies [25, 32] implemented intervention solely on provider-focused interventions while one RCT study used multi intervention components that targeted both HCPs and pregnant women [23]. One of the retrospective studies involved delivering an electronic reminder “best practice alert” within the medical record system by alerting HCPs to offer maternal pertussis vaccination to their pregnant patients [25]. Post-implementation of best practice alert, uptake of pertussis vaccine during pregnancy was significantly improved to 97% compared with 48% of postpartum pertussis vaccination uptake prior to the program. The computed absolute benefit increase (ABI) of the intervention was 49% (95% CI 48% to 50%) (Table 3). Healy et al. [32] evaluated an American College of Obstetricians and Gynaecologists (ACOG) tool kit that aimed to improve HCPs’ awareness of the recommendation to vaccinate pregnant women with pertussis vaccines. The uptake of pertussis vaccine among pregnant women was significantly improved after the release of the ACOG tool kit in the tertiary care centre. The ABI generated from this study was 25% (95% CI 11% to 37%) (Table 3).

#### Pregnant woman-focused interventions

Two RCT studies [24, 33] evaluated the sole effect of pregnant women-focused interventions alone while three studies also incorporated other intervention components [23, 25, 34]. Kris et al. [24] assessed the effect of two Elaboration Likelihood Model (ELM) based vaccine educational interventions—an affective messaging video and a cognitive messaging iBook intervention among pregnant African American women [24]. Only 6% and 7% received the pertussis vaccination during pregnancy in the iBook and video groups, respectively. Sample sizes were too small to obtain meaningful estimates in the improvement of pertussis vaccination during pregnancy. However, of the two interventions, the iBook was significantly associated with uptake of the postpartum pertussis vaccination compared with women in the control group (Table 3). Payakchat et al. [33] conducted a prospective study among pregnant women who were randomized to receive either the standard CDC pertussis vaccine information statement (sVIS) or a modified version (mVIS). There was no significant differences in the pertussis vaccination uptake during pregnancy between the sVIS and mVIS groups. The computed ABI for the study was 3% (95% CI -7% to 15%) (Table 3).

#### Interventions to enhance access to pertussis vaccination

Our review found no studies that implemented interventions solely focused on enhancing access to the pertussis immunization during pregnancy. However, two of the reviewed studies included strategies to enhance vaccine access along with two of the classified intervention types: pregnant woman-focused and provider-focused strategies [23, 34]. One of the studies was a cluster-randomized trial [23] while the other was a prospective observational study [34].

#### Bundled interventions

The reviewed studies included only two intervention components as part of bundled interventions [23, 34]. Chamberlain et al. [23] introduced multi-component antenatal vaccine promotion package among 11 obstetric practices in Georgia. Each intervention obstetric practice was instructed to hand out iPads pre-loaded with lessons demonstrating the importance of maternal immunization to obstetric patients in examination rooms. Chamberlin et al. [23] also evaluated the use of identification of a vaccine champion and assessed whether stocking of influenza and pertussis vaccines in obstetric practices could improve vaccine uptake during pregnancy. Women who received pertussis vaccination during pregnancy were significantly more likely to have been enrolled from a practice stocking pertussis vaccines than women who did not receive a pertussis vaccine during pregnancy (78% vs 51%; p < 0.01). Overall, antenatal pertussis vaccination uptake was higher in the bundled intervention group than the control group, although improvements were not significant (RR 1.58, 95% CI 0.81, 3.07) [23].

Mohammed et al. [34] aimed to estimate maternal vaccine uptake pre-post introduction of a midwife delivered maternal immunization program at a territory obstetric hospital, South Australia. The midwife vaccine delivery program in South Australia equipped midwives with knowledge and skills to engage with pregnant women on the topic of maternal immunization and administer pertussis immunizations to pregnant women [35]. The adjusted odds of women receiving pertussis vaccination during pregnancy were significantly higher after the implementation of the midwife delivered program compared with women who delivered babies prior to the program (AOR 21.1, 95% CI 6.14–72.9; p<0.001) [33]. The calculated ABI for this study was 70% (95% CI 50% to 82%).

## Discussion

Given the well-documented benefits of maternal pertussis immunization in protecting young infants, our review findings are relevant to HCPs and public health policy makers, to guide the establishment of effective maternal pertussis immunization programs. Our review identified six studies evaluating the effectiveness of interventions that promote pertussis vaccination in pregnant women. These studies primarily focused on interventions targeting either HCPs or pregnant women. Our review included three RCTs and three observational studies. RCTs are the most rigorous scientific method for appraising the effectiveness of health care interventions [36]. The interventions in all the three RCTs included in this review did not demonstrate a significant improvement in the uptake of pertussis vaccination during pregnancy, although two studies failed to attain their sample size estimates.

The three observational studies in our review have reported statistically significant absolute increases in the vaccination rate of at least 25% [25, 32, 34]. Mohammed et al. [34] demonstrated provision of pertussis vaccination by midwives at the place of antenatal service was strongly associated with increased pertussis vaccination uptake during pregnancy. The program enables registered midwives to administer vaccination during pregnancy using a standing medication order, without seeking permission from a referring medical doctor [34]. Previous studies suggested that administering maternal immunizations through standard antenatal care by midwives could improve vaccination uptake among pregnant women [37, 38]. However, the relatively small sample size of the reviewed study could be a limitation to the study findings [34].

Previous studies have shown the implementation of a “best practice alert” with in the electronic medical record is associated with improved uptake of influenza vaccines in several high-risk groups [39–41] which supports our reviewed observational study findings in pregnant women [25]. Installing an automated reminder within electronic medical records in an antenatal care setting may encourage health care provider–patient discussions on the safety, efficacy, and necessity of pertussis vaccination during pregnancy. The use of the best-practice alert would also enable prenatal care providers to administer the vaccine at a moment when the pregnant women can act immediately with a minimum of additional time, effort or cost.

The finding of one of the reviewed observational studies [32] is also consistent with earlier research that multiple educational interventions to improve provider awareness has improved vaccine uptake among pregnant women in antenatal care settings [38, 39, 42]. Several studies have also reported that recommendation from maternity care providers is the most important factor in improving vaccination uptake during pregnancy [43–49]. Many of the barriers cited for pregnant women often apply to HCPs as well, including lack of knowledge about the benefits of maternal vaccinations [50–54]. Pregnant women’s misperceptions about the risk of the disease, effectiveness and safety of vaccination during pregnancy are the main barriers to the delivery of vaccinations during pregnancy [55–58]. Hence, overcoming pregnant women and HCP barriers play a major role in improving pertussis vaccination uptake among pregnant women.

Two of the studies assessing the sole effect of pregnant woman-focused interventions were RCTs and found no significant effect of pregnant woman-focused educational interventions [24, 33]. Although, these studies have shown a positive effect of educational interventions on improving pertussis vaccination uptake among pregnant women, they did not significantly improve uptake of pertussis vaccination during pregnancy. It could be argued that interventions solely focussed on educating pregnant women on the benefits of vaccines might not be an effective strategy. Moniz et al. [59] argued that the content of the message in educational interventions might influence its effectiveness and further studies assessing messaging would be of value. There is a need for high-quality patient education highlighting the role of maternal pertussis vaccination in preventing severe pertussis infection in very young infants. Educational materials on maternal pertussis immunization should also be easily readable and accessible to women from culturally and linguistically diverse backgrounds. There is also a need for studies in other countries and low resource settings as it is likely that interventions will need to be cognisant of cultural considerations. In addition, understanding the psychological and social factors influencing women’s decisions to accept vaccines during pregnancy could help in designing strong maternal immunization programs.

### Limitations

Although our review tried to standardize intervention into distinct classifications to enhance their comparability, some studies included interventions of more than one classification, which complicated comparability between interventions. Some of the studies were not adequately powered and were susceptible to bias and thus may only provide indirect evidence of effectiveness. Vaccination behaviour influences the self-report and explains a tendency to overestimate vaccination coverage in self-reporting compared to the electronic medical record [60]. Hence, the reviewed studies with self-reported vaccination are likely biased toward overestimating the intervention’s effect. Moreover, none of the reviewed studies takes into account the impact of contemporaneous vaccination for influenza as a predictor of pertussis vaccination uptake. In other words, participants could be more likely to be vaccinated for pertussis during the time of year when HCPs were also recommending vaccination for influenza particularly during the flu season. Furthermore, recommended national changes in timing of maternal pertussis vaccination from postpartum to antepartum may have introduced bias in comparison of vaccination coverage between standard care and intervention groups in some of the observational studies. In addition, most of the reviewed studies were done in the US and the difference in access to the antenatal health care system among countries limits the generalizability of the results internationally.

Overall, the certainty of the findings in this review are low. To improve the certainty of evidence more RCTs are required. In situations where only observational designs are feasible, consideration of how best to limit potential bias is paramount. Before and after studies should use at least three data points before and after the implementation of the intervention, and adjust for secular trend in the analysis [61].

## Conclusions

The best available evidence suggests that to improve maternal pertussis vaccination to protect young infants, HCPs should inform all pregnant women about the importance of pertussis vaccination during pregnancy, incorporate midwife delivered maternal immunization program at antenatal clinics, use provider reminder systems to target unimmunized pregnant women, and include maternal pertussis immunization as part of standard antenatal care.

## Supporting information

S1 Table PRISMA checklist. (PDF)

S2 Table Database search strategies. (PDF)

S3 Table Quality assessment of the reviewed randomized controlled trials. (PDF)

S4 Table Quality assessment of the reviewed observational studies. (PDF)

S5 Table Characteristics of the excluded studies. (PDF)
