# Supplementary material for: A systematic review of interventions to improve uptake of pertussis vaccination in pregnancy
Source: PLoS One. 2019 Mar 28;14(3):e0214538. doi: 10.1371/journal.pone.0214538 (PMC6438510; doi:10.1371/journal.pone.0214538)
Supplement: S2 Table — (PDF) [file pone.0214538.s002.pdf]

S2 Table. Database search strategies

PubMed

1. Pertussis vaccine	2. Pregnant women
Pertussis vaccine [majr] OR Pertussis vaccin*[ALL] OR Whooping cough vaccin*[ALL] OR Diphtheria-Tetanus-acellular Pertussis Vaccin* [ALL] OR DTaP Vaccin* [ALL] OR Diphtheria-Tetanus-Pertussis Vaccin*[ALL]	Pregnant women [majr] OR Pregnan*[ALL] OR Maternal* [ALL]

(Pregnant women [MH] OR Pregnan*[ALL] OR Maternal* [ALL]) AND (Pertussis vaccine [MH] OR Pertussis vaccin*[ALL] OR Whooping cough vaccin*[ALL] OR Diphtheria-Tetanus-acellular Pertussis Vaccin* [ALL] OR DTaP Vaccin* [ALL] OR Diphtheria-Tetanus-Pertussis Vaccin*[ALL) NOT (ANIMALS[MH] NOT Humans[MH])

1 AND 2

Hits= 578

Filter= Pub dates 10 years

Hits= 350

PMC

(Pregnant women [majr] OR Pregnant[ALL] OR Pregnancy[ALL] OR Maternal [ALL]) AND (Pertussis vaccine [majr] OR Pertussis vaccin*[ALL] OR Whooping cough vaccin*[ALL] OR Diphtheria-Tetanus-acellular Pertussis Vaccin* [ALL] OR DTaP Vaccin* [ALL] OR Diphtheria-Tetanus-Pertussis Vaccin*[ALL) NOT (ANIMALS[MH] NOT Humans[MH])

Hits =1631

Filter= Pub dates 10 years

Hits= 865

Medline- Ovid

1. exp *Pregnant women/

2. Pregnan$.MP.

3. Maternal$ .MP.

4. 1 or 2 or 3 AND

5. exp *Pertussis vaccine/

6. Pertussis vaccin$.MP.

7. Whooping cough vaccin$.MP.

8. Diphtheria-Tetanus-acellular Pertussis Vaccin$ .MP.

9. DTaP Vaccin$ .MP.

10. Diphtheria-Tetanus-Pertussis Vaccin$.MP.

11 5 or 6 or 7 or 8 or 9 or 10

12. 4 and 11

13 limit 12 to yr= "2006 -Current"

14. Humans/

15. exp animals/

16.15 NOT 14

17. 13 NOT 16

Hits= 1946

CINAHL

#	**Query**
S13	S9 AND S12
S12	(MM "Pertussis Vaccine") OR TX "Pertussis vaccin*" OR TX "Whooping cough vaccin*" OR TX "DTap vaccin*"
S11	(MM "Pertussis Vaccine")
S10	(MH "Pertussis Vaccine+")
S9	(MM "Expectant Mothers") OR TX Pregnan* OR TX Maternal*
S8	(MM "Expectant Mothers")
S7	S2 AND S5
S6	S2 AND S5
S5	(MM "Pertussis Vaccine") OR TX "Pertussis vaccin*" OR TX "Whooping cough vaccin*" OR TX "DTap vaccin*"
S4	(MM "Pertussis Vaccine")
S3	(MH "Pertussis Vaccine+")
S2	(MM "Expectant Mothers") OR TX Pregnan* OR TX Maternal*
S1	(MM "Expectant Mothers")

Hits= 381

Combined hits after removing duplicated articles on Endnotes= 1935

Other Sources

I. European Society for Paediatric Infectious Diseases (ESPID)

Abstracts from 2011- 2016

Hits = 0

II. World Society for Paediatric Infectious Diseases (WSPID)

WSPID 2015 and WSPID 2013 and WSPID 2011

Hits= 0

III. International Congress on Infectious Diseases (ICID)= 0

Total hits = 1935
