# Supplementary material for: A systematic review of interventions to improve uptake of pertussis vaccination in pregnancy
Source: PLoS One. 2019 Mar 28;14(3):e0214538. doi: 10.1371/journal.pone.0214538 (PMC6438510; doi:10.1371/journal.pone.0214538)
Supplement: S3 Table — (PDF) [file pone.0214538.s003.pdf]

Study	Risk of Bias						GRADE - Level of evidence			
	Random sequence generation reporting	Allocation concealment	Blinding of participants & outcome assessment	Incomplete outcome data	Selective Reporting	Other bias	Inconsistency	Indirectness	Imprecision	Quality of evidence
Kriss [24]	Quote:<br>"A master database which provided randomization assignments was generated by non-study personnel"<br><br>Judgement<br>Low risk of bias	Investigators enrolling participants could possibly foresee assignment using a list.<br><br>Judgement<br>High risk of bias	Participant: No<br>Assessor: Unclear<br><br>Judgement<br>High risk of bias	15% of the control group and 10% in intervention groups were not included in the analysis<br><br>Judgement<br>High risk of bias	The study did not validate vaccination from vaccination records as described in their protocol on clinicaltrials.gov<br><br>Judgement<br>High risk of bias	Quote: "limited to African American women in a south-eastern metropolitan area"<br><br>Comments:<br>findings may not be generalizable to non-African American populations	No serious Inconsistency	No serious Inconsistency	Insufficient number of participants in both arms (80%power)	Low
Payakachat [33]	Randomly assigned by a coin toss into two groups.<br><br>Judgement<br>Low risk of bias.	Investigators enrolling participants and participants could not possibly foresee assignment.<br><br>Judgement<br>Low risk of bias.	Participants: No<br>Assessor: unclear<br><br>Judgement<br>High risk of bias.	Quote"16 (3%) did not complete the survey due to technical problems with the electronic device"<br><br>Comments: The proportion of missing outcomes compared with observed event risk was not enough to induce relevant bias in intervention effect estimates.<br>Judgement<br>Low risk of bias	Quote "intention to receive the vaccine; and to determine associations between health perceptions with Tdap vaccine receipt"<br><br>Comments<br>The study likely included all pre-specified primary outcomes.	Quote "the majority of patients were of low socioeconomic level and had limited health literacy, the findings have limited generalizability to other pregnant women in different US regions"<br><br>Comments<br>The study may be affected by selection bias.	No serious inconsistency	No serious indirectness	Sufficient number of participants in both arms<br><br>A sample of 250 was required but 291 participants were consented and randomized	Moderate
Chamberlain [23]	Randomisation was achieved using a coin toss by blinded statistician on paired practices.<br><br>Judgement<br>Low risk of bias.	Participants and investigators enrolling participants could possibly foresee assignment.<br><br>Judgement<br>High risk of bias	Participants: No<br>Assessor: Unclear<br><br>Judgement<br>High risk of bias	Quote "Of the 48 women who did not complete the follow-up questionnaire, equal proportions were from the intervention (n = 24) and control (n = 24) groups"<br><br>Comments:<br>The proportion of missing outcomes compared with observed event risk was not enough to induce relevant bias in intervention effect estimates.	The study reports primary and secondary outcomes described by pre-specified criteria on clinicaltrials.gov<br><br>Judgement<br>Low risk of bias	Self-report was the primary method used to judge if a Tdap vaccine was administered<br><br>The study participants may be subjected social desirability bias<br><br>Judgement<br>High risk of bias	No serious Inconsistency	No serious Indirectness	"since the study was designed to detect differences in antenatal vaccine receipt, we likely lacked the sample size necessary to detect significant changes in measures of knowledge, attitudes and beliefs"<br>Comments<br>Underpowered to detect a difference on outcome	Moderate

S3 Table. Quality assessment of the reviewed randomized controlled trials
