# Supplementary material for: A systematic review of interventions to improve uptake of pertussis vaccination in pregnancy
Source: PLoS One. 2019 Mar 28;14(3):e0214538. doi: 10.1371/journal.pone.0214538 (PMC6438510; doi:10.1371/journal.pone.0214538)
Supplement: S4 Table — (PDF) [file pone.0214538.s004.pdf]

S4 Table. Quality assessment of the reviewed observational studies

Quality assessment	Morgan [25]			Healy [32]			Mohammed [34]		
	Yes	No	Unclear	Yes	No	Unclear	Yes	No	Unclear
1. Is it clear in the study what is the cause' and what is the 'effect' (i.e. there is no confusion about which variable comes first)?	Y			Y			Y		
2. Were the participants included in any comparisons similar?			U			U			U
3. Were the participants included in any comparisons receiving similar treatment/care, other than the exposure or intervention of interest?			U		N		Y		
4. Was there a control group?	Y			Y			Y		
5. Were there multiple measurements of the outcome both pre and post the intervention/exposure?		N			N			N	
6. Was follow-up complete, and if not, was follow-up adequately reported and strategies to deal with loss to follow-up employed?			U			U	Y		
7. Were the outcomes of participants included in any comparisons measured in the same way?	Y			Y			Y		
8. Were outcomes measured in a reliable way?	Y			Y				N	
9. Was appropriate statistical analysis used?	Y				N		Y		
