# Supplementary material for: A systematic review of interventions to improve uptake of pertussis vaccination in pregnancy
Source: PLoS One. 2019 Mar 28;14(3):e0214538. doi: 10.1371/journal.pone.0214538 (PMC6438510; doi:10.1371/journal.pone.0214538)
Supplement: S5 Table — (PDF) [file pone.0214538.s005.pdf]

S5 Table. Characteristics of the excluded studies

Study (year)	Reasons for exclusion
Donaldson (2015)	No standard care group was available for comparison
Barber (2017)	No intervention component (Adequate prenatal care is not an intervention)
Manzoni (2016)	Ineligible participants: study was not directed to pregnant women only
Celikel (2013)	Pertussis vaccination uptake was not included
Goldfarb (2014)	No standard care or comparator group
Mazzoni (2016)	Ineligible participants: study was not directed to pregnant women only
Bonville (2015)	No intervention component
Maertens (2016)	No intervention component
Kharbanda (2011)	study was directed to postpartum
Bödeker (2014)	No intervention component
